# Can the collection of expired long-lasting insecticidal nets reduce their coverage and use? Sociocultural aspects related to LLIN life cycle management and use in four districts in Madagascar

**DOI:** 10.1186/s12936-017-2053-z

**Published:** 2017-10-10

**Authors:** Ambinina Ramanantsoa, Marta Wilson-Barthes, Rindra Rahenintsoa, Sarah Hoibak, Harilala Ranaivoharimina, Martha Delphine Rahelimalala, Avotiana Rakotomanga, Alyssa Finlay, Joan Muela Ribera, Koen Peeters Grietens

**Affiliations:** 1 Focus Development Agency, Antananarivo, Madagascar; 2 Department of Sociology, University of Antananarivo, Antananarivo, Madagascar; 3 Emmanuel International, Zomba, Malawi; 4 The Global Fund, Geneva, Switzerland; 5 Ministry of Health, Antananarivo, Madagascar; 6 Ministry of Environment, Antananarivo, Madagascar; 7 USAID-Deliver Project, Antananarivo, Madagascar; 8 Division of Parasitic Diseases and Malaria, CDC, Atlanta, USA; 9 Partners for Applied Social Sciences PASS International, Tessenderlo, Belgium; 10 School of Tropical Medicine and Global Health, Nagasaki University, Nagasaki, Japan; 11 Medical Anthropology Unit, Department of Public Health, Institute of Tropical Medicine, Antwerp, Belgium

**Keywords:** Malaria, Recycling, Disposal, Net collection campaign, Insecticide treated nets (ITNs), Long lasting insecticide treated nets (LLINs)

## Abstract

**Background:**

There is growing awareness of the likely impact increased numbers of LLINs will have on the environment, if not disposed of or recycled appropriately. As part of a World Health Organization (WHO) and United Nations Environment Programme (UNEP) pilot study to assess environmentally-sound and cost-effective LLIN recycling strategies, the USAID-Deliver Project collected 22,559 used bed nets in Madagascar. A social science study was conducted to provide data on socio-cultural factors related to collection and replacement of LLINs, including impact on primary and other net uses.

**Methods:**

Ethnographic exploratory research was carried out following the pilot USAID-Deliver net collection and recycling campaign in Betioky, Tsihombe, Fenerive Est and Ambanja districts of Madagascar, triangulating participant observation, interviewing and group discussions. Sampling was theoretical and data analysis was a continuous and iterative process concurrent to data collection. Final analysis was conducted using NVivo10.

**Results:**

The following themes emerged as contributing to the success of collecting expired LLINs in the community for recycling purposes: (i) net adequacy and preference: characteristic differences between collected and newly distributed nets lead to communities’ reticence to relinquish old nets before confirming new nets were appropriate for intended use. Where newly distributed nets failed to meet local requirements, this was expected to increase alternative uses and decrease household turn over. (ii) Net collection strategies: the net collection campaign brought net use out of the private sphere and into the public arena. Net owners reported feeling ashamed when presenting damaged nets in public for collection, leading to reduced net relinquishment. (iii) Net lifecycle: communities perceived nets as being individually owned and economic value was attributed both to good-condition nets for sleeping and to worn nets for alternative/secondary purposes. Collecting nets at the stage of waste rather than at their prescribed end of life was locally acceptable.

**Conclusion:**

The collection of LLINs for recycling/disposal can lead to lower coverage under certain conditions. Collecting used LLINs may be appropriate under the following conditions: (i) nets are collected at the stage of waste; (ii) new nets are in line with community preferences; and (iii) collection strategies have been agreed upon within the community prior to replacement activities. Any collection/recycling of old LLINs should be based on in-depth understanding of the local context and include participatory processes to prevent reduced coverage.

**Electronic supplementary material:**

The online version of this article (doi:10.1186/s12936-017-2053-z) contains supplementary material, which is available to authorized users.

## Background

The WHO’s Global Malaria Programme (GMP) recommends universal coverage with long-lasting insecticidal nets (LLINs) for all populations at risk of malaria in areas targeted for prevention and control [1]. Coupled with increased distribution capacity and decreasing cost, global access to LLINs has increased by more than 50% in the past decade [2], with 60% of the population in sub-Saharan Africa [95% CI 57–64%] now having access to ITNs in their household [3]. In the past 2 years more ITNs were delivered in this region—189 million in 2014 and 178 million in 2015—than in any prior years [3]. LLINs continue to be regarded as the most effective control measure against malaria [1–4]. Nets treated with insecticides are more effective in reducing mortality and morbidity when compared to sleeping under no net or untreated nets [4–7]. LLINs are cost-effective in reducing transmission, amounting in 2013 to an estimated $2.5 billion savings in malaria treatment costs [2, 8, 9]. In sub-Saharan Africa, LLIN use contributed the largest savings to malaria case management costs (68%) between 2001 and 2014 [10].

LLINs however, do not last forever. While they are understood to have a significantly longer protective effect compared to conventional, untreated or manually treated nets, their durability and level of insecticide deteriorates with time, use and maintenance. Long-lasting insecticidal nets, as recommended for public health use by the World Health Organization Pesticide Evaluation Scheme (WHOPES), have a manufacturer recommended lifespan of 3–5 years [11, 12] compared to conventional nets which require annual retreatment [1]. Despite this extended lifespan, LLINs require timely replacement for continued effectiveness in malaria prevention.

With millions of insecticide treated nets (ITNs) and LLINs already distributed, and knowing that these nets require replacement every 3 years [3], a dilemma emerges from the potential environmental effect of the growing mass of discarded LLINs. The amount of polymer in one LLIN is equivalent to approximately 40–50 plastic bags or roughly 500 g of plastic [13, 14]. The global distribution of over 659 million bed nets by 2015 [15] therefore translates into a potential waste stream of over 216,000 metric tons of homogeneous polymer. An additional concern is that up to 30–80% of the full beginning-of-life dose of pesticide may remain in impregnated nets that are up to 7 years old [13]. While insecticide-treated plastics can be incinerated safely in high-temperature furnaces, suitable facilities are lacking in most countries. Burial away from water sources and preferably in non-permeable soil is an appropriate disposal method for net bags and old LLINs in the absence of a suitable high-temperature incinerator, though such disposal practices are not commonly adhered to in most malaria-endemic regions [16].

Responding to this environmental concern, the UNEP and WHO *Strategic Approach to International Chemicals Management (SAICM) Quick Start Programme Trust Fund* conducted a pilot project in 2010–2011 in Madagascar, Kenya and Tanzania to identify and assess the feasibility of environmentally-sound and cost-effective options for the collection, recycling and disposal of expired LLINs [17]. In all country sites, a social science study was carried out to provide exploratory data on local socio-cultural factors that might prove decisive for the effective management of used LLINs and related consequences for uptake and net use. This article presents findings from the qualitative study carried out in four districts in Madagascar, with the objective to provide preliminary data on the influence of socio-cultural and ethical factors on LLIN coverage and use in the community, and the implication of these factors for future net collection activities.

## Methods

### Study design

Ethnographic methods, representing an emergent theory design, were employed to assess the LLIN life cycle, alternative net uses and other socio-cultural issues related to the collection, disposal and recycling of nets at community level. The anthropological study was ancillary to the USAID-Deliver recycling campaign carried out in Betioky, Ampanihy, Beloha, Taolagnaro, Tsihombe, and Ambovombe districts to assess the viability of collecting and recycling LLINs. A total of 22,559 nets were collected [13]. Having undergone collection campaigns, the Madagascar setting presented a unique opportunity to explore barriers, enabling factors to collection of nets and related implications of these campaigns in the community.

### Study site and population

The study employed a theoretical, non-random cluster sampling method within which communities were selected based on the criteria required to address the study objectives (Table 1). Four districts were purposefully selected: Betioky, Tsihombe, Ambanja, and Fenerive Est. Criteria for inclusion were collection campaign participation and success rates, district size, district geography and climate, epidemiological indicators, and accessibility to researchers. Betioky and Tsihombe were selected because of their inclusion in the USAID-Deliver recycling campaign and because of the wide variation in collection success rates (61 and 15% respectively), providing a strong basis for inter-district comparison. Ambanja and Fenerive Est were outside the USAID-Deliver programme. Locations within the districts were theoretically selected (Table 2).

**Table 1:** Study objectives by factor

Exploratory factor	Research objective
Net coverage	To evaluate whether, in real life conditions, the presence of the old nets in the household is likely to maintain, increase or decrease general net coverage and use of LLINS
Alternative/secondary net use	To assess under what conditions LLINs are no longer used for sleeping
	To identify alternative uses for bed nets which are no longer used for sleeping
	To inform the decision, based on observed socio-cultural and ethical activities, of whether or not alternative net uses should be discouraged or encouraged for increased malaria prevention
LLIN distribution effectivity	To assess the factors related to continuation or cessation of using old ITN/LLINs for sleeping when newer nets are introduced in the household (i.e. do pregnant women and children use new nets or continue to use old nets while saving new nets for future use)
Recycling practices and perceptions	To assess community acceptability of returning bed nets which are no longer used for sleeping during collection campaigns
	To explore general community perceptions about reusing, recycling, energy recovery, replacing and disposing of LLINs
Perceived health risks from recycled LLINs	To assess communities’ perceived risk of using LLINs after recycling campaigns and related IEC campaigns attributed to the “toxicity” or “harmfulness” of LLINs
	To gather preliminary data on any possible concern related to the future implementation and corresponding health risks of LLIN life-cycle management
Net type	To describe in detail the life cycle of varying types and make of bed nets in communities
	To assess community practices of LLIN use, maintenance and disposal in relation to net type and characteristics, both for nets currently in use and those newly distributed
Net waste	To evaluate what socio-cultural and physical characteristics define a net as “expired” or “waste” (i.e. no longer used for any purposes)
	To identify local communities’ current methods of LLIN disposal of nets which are no longer used for any purposes

**Table 2:** Characteristics and demography of study districts

	Betioky	Tsihombe	Ambanja	Fenerive Est
Population	194,562	104,369	180,446	292,219
Ethnic group (predominant)	Mahafaly	Antandroy	Antakarana; Sakalava	Betsimisaraka
LLIN collection through USAID-Deliver (%)	61	15	None collected	None collected
Municipalities	27	13	23	12
Fokontany	342	77	187	150
Geography	South-west	South	Northwest	East seaside area
		Sub-desert	Diana region	
		Seaside area	East seaside area	
Topography				
Latitude	23° 38 south	25° 19 south	13.68° south	17° 22 south
Longitude	44° 55 east	45° 29 east	48.45° east	49° 25 east
Malaria transmission seasonality (facies) 2010	Sub-desert	Sub-desert	Equatorial	Equatorial
	Occasional	Occasional	January–June	January–June
	Sporadic transmission	Sporadic transmission	Long-lasting transmission	Long-lasting transmission
Malaria prevalence (2010) (%)	1.30	3.74	12.04	11.33
Accessibility	Difficult	Difficult	Difficult	Highly accessible

Population data from National Statistics Institute of Madagascar (INSTAT/DDSS 2011)

### Data collection

Three qualitative data techniques were triangulated: formal and informal interviewing, focus group discussions (FGD), and direct observation. Information collected through informal discussions was used to guide participant selection for formal interviews and focus group discussions. Participant observation was used to compare interview statements with directly observed actions and was informed by an adaptive observation guide.

#### Interviews

Informants were selected based on gender, age, religion, ethnicity, locality, socio-economic status, net possession, net use, net preference, and opinions on net recycling. Formal interviews were conducted at both the household (community) and authoritative level. Authorities selected for formal interviews were expected to be involved in local health services or health surveillance.

#### Focus group discussions

Focus group discussions (FGD) have been proven as a critical tool for allowing participants to recall and express ideas that might otherwise not have emerged when alone [18] and serve to provide supplementary information to individual in-depth interviews [19]. Focus group discussions were held in groups of 3–10 people and followed an adaptive discussion guide. Each FGD sought to address only a select number of the total study objectives. Participants were recruited through matching of socio-demographic criteria to the study objective under discussion in the given group. Natural group discussions, defined as discussions with “persons who know each other already” [20], were held with key malaria stakeholders who were involved in policy decisions surrounding LLIN use and recycling at the local level, and with selected community members.

#### Participant observation

The observation of people’s behaviour in their natural setting is a fundamental and often neglected part of qualitative research. It enables comparison of people’s ideas and stated behaviour with their actual behaviour. Participant observation was conducted within and outside district households in order to contrast people’s stated behaviour in interviews and FGDs with their daily actions. All investigators relied on an observation guide to track and record observations.

### Sampling

After identifying district locations, respondents were theoretically selected (i.e. based on emergent results). Key informants were characterized under “authority” or “community” status (Additional file 1: Table 3). This breakdown served to guide a varied distribution of respondents from different standings in the community and to better inform respondent experience with net usage and distribution. All collected data were recorded after gaining participants’ verbal consent.

### Analysis

Data analysis was a continuous, flexible and iterative process concurrent to data collection. All interviews, focus groups, and informal discussions were held in Malagasy, electronically recorded, transcribed and translated into French. Data was analysed in NVivo 10 developed by QSR international. A record of the most relevant informal conversations and important observations in the field were included in the database until saturation of results was achieved. Discussion between investigators was recorded and also included in the database. Intermittent data analysis in NVivo was conducted after data collection was complete in each district and cumulatively at the end of the study.

### Concept definitions

*Alternative net use* is defined as the use of a net for another purpose than sleeping even when in good condition for sleeping. *Secondary net use* is used for a net that is no longer acceptable for sleeping and is re-purposed for other domestic and economic uses. *Waste*: a (piece of) used net that cannot be useful for sleeping, nor any alternative or secondary purposes. *Fokontany:* the smallest administrative division in Madagascar.

### Ethical considerations

The study was reviewed by WHO and received a waiver from the Ministry of Health ethics committee in Madagascar. All fieldwork followed the code of ethics of the American Anthropological Association (AAA) [21]. Following the AAA’s 1986 Statement on ethics for principles of professional responsibility, all interviewees were informed before the start of the interview about project goals, question topic, their right to refuse or stop an interview at any point, their right to withdraw any information during or after the interview, and the intended use of the results for scientific publications and reporting. Oral consent was preferred, since interviewees were not at risk of physical or psychological harm and because written consent during conversation presents a potential reason for mistrust. All data was anonymized and labelled by study code to ensure participant anonymity.

## Results

Research was conducted in 2011 for 2 month periods per each district: from May to June in Betioky, from June to July in Tsihombe, from October to November in Fenerive Est, and from November to December in Ambanja.

### Study participants

Sixty-eight formal interviews (FI), 18 focus group discussions (FGD), 13 direct observations and 10 informal interviews (II) were conducted in the Betioky, Tsihombe, Fenerive Est, and Ambanja districts of Madagascar (interview typology is presented in Additional file 1: Table 3). Based on the emergent findings from this study, the collection of expired LLINs for recycling/disposal had the potential to lower net coverage and use in the community due to the following influencing factors:

#### Net adequacy

All households in the four study sites had at least one LLIN in use, with the majority of households possessing a second LLIN for additional family members, guests or use outside the home, such as working in the fields or travel. Household members in all districts obtained their household nets primarily through distribution campaigns or donation and, in a minority of cases, purchased polyester nets or non-treated nets at local markets. Some participants of formal interviews in Betioky and other districts said they felt one net for every two people would be a better standard compared to the current ratio of one net for every three people, the standard set by the Ministry of Health and distributing partners in the most recent campaign. Participants explained they felt this ratio of one net per two persons was reasonable as some family members often could not sleep together for cultural reasons, such brothers and sisters or daughters with a single father. Insufficient coverage and unequal distribution of LLINs was reported periodically by household members across the four study sites and related directly to the level of willingness to give up nets for collection. In several cases during the 2010 distribution campaign only one net was distributed per household. In districts that participated in the USAID-Deliver campaign, new nets were often reported to have been unequally distributed. FGD’s in Betioky and Tsihombe described large households receiving fewer nets than smaller households due to lack of planning and inadequate resources. Community agents and *fokontany* leaders, especially those of rural Tsihombe, cited nonpayment and inadequate transportation as reasons distribution activities were halted. Formal interviews with authority members gave accounts of having insufficient quantities of new nets to distribute in proportion to the number collected. Consequently, in all districts the following trends were observed: (i) reluctance to turn over old nets due to a fear new nets would not be provided; (ii) overall preference for distributing a new net at the same time old nets were collected; and (iii) willingness to receive new nets prior to relinquishing old nets.

Net use was motivated by (i) the experienced comfort (including sleep without interruption from mosquitoes and other bugs) and (ii) the perceived health protection the LLIN provided. Routine use varied also by gender and age of the user. Participants in FGDs conveyed that women and children under five were the predominate population to sleep under LLINs on a nightly basis.

Sleeping without a mosquito net was a practice predominately associated with the virility and household authority of men (especially in Fenerive Est and Ambanja districts) even for those who had had previous malaria infections. Similarly, adolescents (i.e. age 6–14 years) conveyed an increased likelihood of sleeping without LLINs. Adolescent males, at a certain stage, reported leaving their parents’ bed and sleeping in an alternative location in the house. This was seen predominately in Tsihombe, where they were considered “old enough to be able to fend off the mosquitoes that bite them at night” (informal interview—Tsihombe). Children were left unprotected when family members needed to bring the household LLIN to the fields during the farming season.

#### Net preference

Community members stated that net use depended largely on whether or not they owned their preferred type of net. Net preference depended on net texture, mesh size, overall net size, and the presence or lack of a built-in opening (“*varavarana”*). In all districts, polyester nets with an opening were preferred to polyethylene nets. Of the possible brands of LLINs in production, Permanent® and Interceptor® polyester nets with a built-in opening were preferred across the four districts while polyethylene nets like Olyset® brand were least preferred brand. Polyester net preference was based on: (i) the soft texture, which participants said facilitated easier storage and did not leave any indentation on the face or body during sleep; (ii) the presence of a built opening in some net models—in select cases members were observed to tear openings when one did not already exist (e.g. Tsihombe); (iii) large overall net size, with larger rectangular nets allowing total bed coverage, which was preferred by members in more populated regions where beds were valued as furniture; (iv) smaller mesh size which deterred insect penetration; and (v) the real or perceived durability compared to polyethylene nets.

Because of the preference for these net characteristics, mothers and children chose to sleep under polyester nets even when they were past their 3-year life cycle and even when new polyethylene LLINs of other models were available. Study participants were consequently less eager to give their polyester nets during collection campaigns when they were unsure about receiving the same type of net during distribution.

#### Net life-cycle

In all districts, the length of the net’s life cycle was defined by its physical condition and economic value. The life of LLINs for sleeping was locally perceived to wane as early as 6 months with the first visible hole or tear. While LLINs are considered to have a 3-year life cycle, people reported to see the nets’ economic and social value to last for much longer based on the nets’ alternate and secondary value. Most commonly reported types of alternative and secondary uses were to use nets for fishing, to use as blankets, to pack farmed produce, to catch locusts or as a household ornament (e.g. curtains). Other less commonly reported uses included protection from the sun, sanitary towels, toilet paper, and rope to tie livestock and ox carts. Nets with too many holes or tears to be able to be sewn together were qualified as expired. Direct observation showed nets that had reached the point of complete waste were predominately left abandoned, as well as also thrown in rivers, burned or buried. In the urban communities of Fenerive Est and Ambanja, collected waste was dumped in a landfill away from the city, or in a designated waste spot by the seaside.

#### Net-collection strategy

Community members and authorities in all districts were asked about their experience and opinion with four possible collection strategies: (i) simultaneous collection during distribution campaigns; (ii) net collection at basic health centers (BHCs); (iii) household collection through door-to-door outreach; and, (iv) collection at public arenas including grocery and retail outlets.

Collection strategy and duration varied between the two districts included in the deliver campaign (Betioky and Tsihombe), which contributed to the discrepancy in collection rates in these districts (Table 2). In Tsihombe, community agents responsible for net collection stated the “cascade” training they received, defined as informal briefings of lead community workers, lasted only a few days and was “insufficient.” In Betioky, net collection leaders were trained via radio outreach and formal in-person sessions. In this district, community agents followed a door-to-door collection strategy while in Tsihombe nets were collected passively, with community members being responsible for bringing worn nets voluntarily to public arenas including health centre and markets. Participants of direct interviews and FGDs in Tsihombe stated a lack of information was provided to household and community members about (i) the timing of net collection and (ii) the intent and purpose of collection (FGD—Community Health Agents, Tsihombe). Community members in both districts stated voluntary health workers and local *fokontany* authorities had a lower interest in becoming involved in net collection activities because campaigns were directed by a private international organization which controlled worker payment and was unfamiliar to the community and authority members.

Members in districts outside the USAID Deliver programme (Ambanja and Fenerive Est) conveyed they understood there was an increased risk of failure to turn in obsolete nets if new nets were distributed before collection. Community members in these districts believed incentives would be an effective tool for encouraging members to turn in old nets (FGDs—Ambanja and Fenerive Est). Suggested incentives included rice, oil, beans and monetary compensation.

Community members in both collection and non-collection districts conveyed the following trends: (i) preference for door to door collection campaigns based on difficulty accessing BHCs and public markets; (ii) desire for routine collection campaigns so that net owners can plan for receiving new nets; (iii) and ineffectiveness of campaigns carried out at markets and grocery/retail outlets due to the establishments’ emphasis on sales and difficulty of access for community and *fokontany*. Where nets were collected at markets, retail stores, or health centers, this brought net use out of the private and into the public sphere. A sense of embarrassment was associated with returning worn nets to these public spaces, with community members reporting feeling ashamed by having to present dirty, ripped or bad smelling nets in public for collection. Women in Ambanja (FGD) however expressed a strong preference for collection activities to be carried out at basic health centres (BHCs). This provided women an opportunity to access additional health services and to avoid the “nepotism” of certain *fokontany* chiefs they often experienced during net distributions/replacement.

#### Concept of recycling

At all study sites recycling of any waste was a new concept for community and authority members. Recycling was often confused with using LLINs for alternative uses rather than the direct disposal and reprocessing of raw materials, especially at the community level. After members understood the definition and implications of recycling, those interviewed expressed support for net collection campaigns, stating their first choice was to trade in nets at their end-of-life cycle for new nets of the same make. There was no cultural objection to net collection if new nets were guaranteed. Messaging around net use, maintenance and package handling had been communicated by the pilot collectors to communities during the campaigns in Tishombe and Betioky. Unlike the variation in effectively communicating the collection strategy between the two districts, messaging on net use and maintenance appeared to have been better understood. Members were able to describe the importance of washing nets regularly—“every 2 months” or “every few months” (informal interviews—Betoiky) and disposing of packaging in a safe way, “it should be burnt, kept away from food and from the reach of children” (community member—Tishombe).

#### Perceived health risks related to insecticides

A small minority of participants expressed fear of the potential health risks remaining pesticides might pose in the recycled products derived from LLINs. Focus group participants in Tsihombe voiced apprehension that recycled nets that came into direct contact with food (as when used in bags for sweet potatoes) would be “fatal”. Community men in Ambanja (informal discussion) expressed some fear of health hazards from direct contact between recycled products and skin. Also from messaging of proper net use and handling, members of a FGD in Tishombe vocalized, to a lesser extent, the health risks of using LLINs for alternative purposes chiefly fishing, though this communication was not supported by actions observed through direct observations.

## Discussion

Collecting LLINs at their end of life cycle has the possibility to reduce net coverage and use in the community if social and cultural factors are not well understood and integrated into campaigns. In Madagascar, preference for certain net characteristics and reluctance to exchange nets due to remaining economic value placed on nets for their primary, secondary and alternative use were further deterrents to relinquishing nets for collection. The influence of LLIN preference on optimal net use and coverage has been demonstrated elsewhere. In the Solomon Islands community members exhibited a preference for polyester nets, citing large mesh size and inadequate bed coverage of polyethylene nets as reasons for intermittent or no net use [22]. Research of LLIN use in Peru similarly found mesh size and transparency of polyethylene nets to lead to lower net use compared to traditional opaque nets [23]. Net preference, as based on net make, size, and colour, has been found to influence LLIN total coverage in multiple other settings in sub-Saharan Africa [24–26]. Programmes seeking to increase net coverage and appropriate net use must account for LLIN preference of the local community.

The economic value placed on nets, for both sleeping and alternative uses, along with the sense of individual net ownership, raises the question whether it is feasible to recycle LLINs at their end of life considering they are being used far longer and for purposes other than sleeping. Collecting nets at the stage of perceived “waste” conversely—when they are no longer used for any alternative or secondary purpose and no longer hold economic value would constitute a more realistic and acceptable approach at the local level.

The strategy by which expired LLINs are collected from communities influences how likely members are to relinquish worn nets and maintain total net coverage with new LLINs. For a collection strategy to be effective, early involvement from both local authority and community members is necessary. Informing district participants of collection intent and methods *prior to* upcoming distribution campaigns can serve to limit feelings of betrayal or infringement on net ownership when nets are collected and exchanged for new ones. In this setting, door-to-door net collection represents the most effective collection strategy, evidenced by the high rate of nets collected through this method in the Betioky district and by the stated preference for this strategy across all study districts. As worn and dirty nets can raise issues of social stigmatization and embarrassment, door-to-door campaigns can serve to avoid feelings of shame by leaving collection activities in the private sphere instead of public arenas. The ability of community members to retain and verbalize messages they learned during collection campaigns around proper net use, maintenance and handling indicates that educational communication efforts could be effective in presenting applications for alternative net uses to communities. Because discrepancies were observed between verbal discussions of net usage and actual daily use practices such as for fishing and food storage, additional measures, beyond education and communication campaigns, must be incorporated in any net replacement/distribution campaigns.

For any LLIN collection strategy to be effective in removing end-of-life nets from the community without reducing coverage, it must (i) ensure users are confident that they will receive a new net at the time of collection and (ii) that the collection strategy has been previously explained and agreed upon within the community to overcome barriers associated with relinquishing privately owned items. Taking into account these provisions at the local level, the effectiveness of any net collection strategy could raise ethical concerns if users are not informed that relinquishing LLINs is voluntary and cannot be required by authority leaders or governing entities.

An effective collection strategy must also consider the timing of when new LLINs are distributed in relation to when worn nets are collected. New LLINs must be distributed at the same time worn nets are collected to prevent gaps in coverage. While net preference may hinder a community’s willingness to relinquish nets for fear of receiving an “inferior” net type, it is not possible for countries to procure, or for donors to provide, nets based on this preference [27, 28], due largely to cost-effectiveness of distribution and supply chain limitations. Limitations on a country’s ability to meet the net preferences of its population increase the importance of efficient net replacement strategy and of educating communities on appropriate of net use.

It is possible that the collection of LLINs that are being used for any purpose might under certain conditions lead to lower LLIN coverage. While coverage rates are shown to be higher for vulnerable populations [29], net possession does not necessarily indicate utilization [30], as alternative or misuse of LLINs continue to be cited as one of the reasons why nets are not used for sleeping [22, 31–33]. It is possible that low social acceptability of worn nets or of non-preferred new nets leads to alternative uses [23], thereby reducing coverage. If residual insecticide is significant in expired LLINs still in secondary use, the health impact may be significant based on the type of repurposing. Expired LLINs which are employed for alternative vector control uses, such as using the net as eves covers, doors or bath curtain [17, 34] may be worthwhile to advocate as a malaria prevention strategy. However, if expired LLINs are being used for non-vector control purposes, such as fishing and in crop protection, [25, 26] potential health impact of residual insecticides needs to be understood in greater depth to dissuade potentially harmful practices. There is a need to further document such alternative uses and to clarify the extent to which new or expired nets are being put to secondary use.

### Study limitations

The study was limited in scope to a qualitative design and would have benefitted from an additional quantitative strand to quantify assessed factors and their relation to coverage and use of LLINs. The depth of information gathered through formal interviews and from focus groups was at times limited by social desirability bias manifesting in participants’ fears to discuss the use of nets for alternative or secondary purposes as this was often considered to be socially unacceptable or even be breaking the law.

## Conclusion

There is a risk that collecting LLINs for the purpose of recycling or disposal may decrease net coverage and use in the community. As no country has yet achieved the WHO recommendation of universal coverage with LIINs in at risk populations, [2, 3] maintaining and increasing coverage rates remains the priority when considering net distribution and collection activities. Net collection and recycling must be part of an integrated waste management after an in-depth analysis of best collection strategies has been conducted in the local context.

## Additional file

**Additional file 1: Table 3.** Typology of interviews and focus group discussion by participant type and district.
