# Supplementary material for: Can the collection of expired long-lasting insecticidal nets reduce their coverage and use? Sociocultural aspects related to LLIN life cycle management and use in four districts in Madagascar
Source: Malar J. 2017 Oct 10;16:404. doi: 10.1186/s12936-017-2053-z (PMC5634957; doi:10.1186/s12936-017-2053-z)
Supplement: Supplementary file 1 — Additional file 1: Table 3. Typology of interviews and focus group discussion by participant type and district. [file 12936_2017_2053_MOESM1_ESM.pdf]

Table 3. – Typology of Interviews and Focus Group Discussion by participant type and district

	In-Depth Formal Interviews (FI)					Focus Group Discussions (FGD)					
	Community		Authority			Community				Authority	
	Women	Men	Community	Administrative	Traditional	Women	Men	Mixed	Community Agent	Chief of Fokontany	Chief of BHC
**Betioky**	**8**	6	4	3	-	3	1	1	-	1	-
**Tsihombe**	8	4	6	3	-	2	1	-	2	1	-
**Ambanja**	5	3	1	1	1	1	1	-	-	-	1
**Fenerive Est.**	8	1	2	4	-	1	1	1	-	-	-
**Total**	29	14	13	11	1	7	4	2	2	2	1

BHC= Basic Health Center
FGD's were comprised of 2 or more people.
